# Distinct Features of Auditory Steady-State Responses as Compared to Transient Event-Related Potentials

**DOI:** 10.1371/journal.pone.0069164

**Published:** 2013-07-09

**Authors:** Li Zhang, Weiwei Peng, Zhiguo Zhang, Li Hu

**Affiliations:** 1 Key Laboratory of Cognition and Personality (Ministry of Education) and School of Psychology, Southwest University, Chongqing, China; 2 Department of Orthopaedics and Traumatology, The University of Hong Kong, Hong Kong, China; 3 Department of Electrical and Electronic Engineering, The University of Hong Kong, Hong Kong, China; Université catholique de Louvain, Belgium

## Abstract

Transient event-related potentials (ERPs) and steady-state responses (SSRs) have been popularly employed to investigate the function of the human brain, but their relationship still remains a matter of debate. Some researchers believed that SSRs could be explained by the linear summation of successive transient ERPs (superposition hypothesis), while others believed that SSRs were the result of the entrainment of a neural rhythm driven by the periodic repetition of a sensory stimulus (oscillatory entrainment hypothesis). In the present study, taking auditory modality as an example, we aimed to clarify the distinct features of SSRs, evoked by the 40-Hz and 60-Hz periodic auditory stimulation, as compared to transient ERPs, evoked by a single click. We observed that (1) SSRs were mainly generated by phase synchronization, while late latency responses (LLRs) in transient ERPs were mainly generated by power enhancement; (2) scalp topographies of LLRs in transient ERPs were markedly different from those of SSRs; (3) the powers of both 40-Hz and 60-Hz SSRs were significantly correlated, while they were not significantly correlated with the N1 power in transient ERPs; (4) whereas SSRs were dominantly modulated by stimulus intensity, middle latency responses (MLRs) were not significantly modulated by both stimulus intensity and subjective loudness judgment, and LLRs were significantly modulated by subjective loudness judgment even within the same stimulus intensity. All these findings indicated that high-frequency SSRs were different from both MLRs and LLRs in transient ERPs, thus supporting the possibility of oscillatory entrainment hypothesis to the generation of SSRs. Therefore, SSRs could be used to explore distinct neural responses as compared to transient ERPs, and help us reveal novel and reliable neural mechanisms of the human brain.

## Introduction

The presentation of a transient sensory event would disturb the spontaneous electroencephalographic (EEG) activity and evoke the event-related potentials (ERPs) that are time-locked and phase-locked to the sudden onset of the sensory stimulus [1], [2]. As one of the most widely-used non-invasive neurophysiologic approaches to study the functions of the human brain, transient ERPs are normally identified in the time domain as a series of monophasic deflections, which are characterized by their polarity, latency, amplitude, and scalp distribution [1], [3]. When presenting a long-lasting periodic repetition of a sensory stimulus, the evoked brain responses attained a steady-state regime (termed steady-state responses, SSRs), in which the amplitude and phase of the constituent frequency features are approximately constant over time [4]. SSRs are composed of a series of identical/similar temporal waveforms, and are normally identified in the frequency domain as peaks appearing at the frequency (and/or its harmonics) of the repeated stimulus, which are characterized by their power and scalp distribution [5]. For this reason, SSRs can be quantified unequivocally as compared to the transient ERPs, and capture several important advantages as summarized in Colon et al [6]. Most importantly, SSRs exhibit a high signal-to-noise ratio (SNR), indicating that a shorter time of data collection is needed to obtain reliable signals [6]–[8].

Although transient ERPs and SSRs have been popularly employed by physiologists, psychologists, and physicians for their respective applications [9]–[13], their relationship remains a matter of debate [5]. While many researchers believe that SSRs are simply the result of the linear summation of successive transient brain responses evoked by the long-lasting periodic repetition of a sensory stimulus (superposition hypothesis) [14]–[18], others support the concept that SSRs are attributed to the stimulus-driven entrainment of an oscillatory network of neurons driven by the periodic repetition of a sensory stimulus (oscillatory entrainment mechanism) [19]–[23].

The superimposition hypothesis, which assumes that the SSRs result from the same neural activities that underlie the transient ERPs, however overlap in time and space, has been supported by findings of strong resemblance between both types of evoked responses [14]–[18]. Specifically, to support the superposition hypothesis, it has been reported that the auditory SSRs evoked by stimulation of 40 Hz could be largely explained by the linear sum of transient auditory ERPs, e.g., auditory brainstem response and MLRs [14], [17], [24], and the visual SSRs elicited by a checkerboard stimulation reversing at different rates can be explained by the temporal superposition of transient visual ERPs [15].

This superposition hypothesis was challenged by the oscillatory entrainment hypothesis, which supported the idea that SSRs were the result of the entrainment of a neural rhythm driven by the periodic repetition of a sensory stimulus [19]–[21]. In this view, SSRs would reflect the ability of neurons to oscillate at particular frequencies (resonant frequencies) coded by the external periodic stimulation [6], [8], thus contributing to a markedly great magnitude of SSRs evoked by a sensory stimulus at the resonant frequencies compared with their adjacent frequencies. For example, a clear peak appearing at 10 Hz and 40 Hz was frequently reported in visual and auditory SSRs in their respective spectra [17], [21], [25]. According to the oscillatory entrainment hypothesis, the neural activity captured by SSRs could be significantly different from that reflected in transient ERPs [4], and they may be generated from distinct neural sources (e.g., the transient auditory ERPs and SSRs were generated from different sources in the auditory cortex) [22], [23]. Indeed, the coexistence of superimposition of transient ERPs and synchronized oscillations has been suggested as an alternative explanation to the generation of SSRs [5]. Specifically, SSRs may be mainly generated from the superimposition of transient ERPs elicited by the stimulus at a given frequency, while reflecting the oscillatory entrainment driven by the stimulus at another frequency [5].

Clarifying the relationship between SSRs and transient ERPs is important to reveal their functional significance in both basic and clinical studies. Taking auditory modality as an example, we aimed to (1) test whether SSRs can be explained by the linear superposition of transient ERPs or the oscillatory entrainment of neurons, resonating at the frequency of stimulation, and to (2) explore, whether the SSRs reflect, at least partially, neural activity, which is distinct from transient ERPs. The temporal, spectral, and spatial characteristics of transient auditory ERPs elicited by a single click were explored and compared with those of SSRs evoked by 40-Hz and 60-Hz periodic stimulation.

## Materials and Methods

### Subjects

Nineteen normal-hearing healthy subjects (10 females), aged between 19 and 24 years (21.9±1.6, mean ± SD), took part in the study. All subjects gave their written informed consent and were paid for their participation. The local ethics committee of Southwest University (Chongqing, China) approved the procedures, which were in accordance with the standards of the Declaration of Helsinki.

### Auditory Stimulation

**Figure 1:**
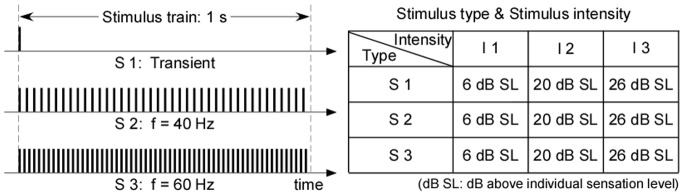
Transient and rapid periodic auditory stimulation. Transient auditory stimuli are 1-ms duration clicks, and rapid periodic auditory stimuli are trains of 1-ms duration clicks that vary in rate of presentation (40 and 60 Hz). The duration of the click train is 1000 ms for both 40-Hz and 60-Hz stimuli. Each type of stimuli (S1: transient; S2: 40-Hz periodic; S3: 60-Hz periodic) are presented at three different intensities (I1 = 6 dB SL, I2 = 20 dB SL, and I3 = 26 dB SL) and delivered through headphones.

Auditory stimuli were sound pulses with 1-ms duration, delivered binaurally through headphones. The sound pulses were presented in three types: single pulse (S1: transient stimuli), 40 consecutive auditory pulses with a repetition rate of 40 Hz (S2: 40-Hz rapid periodic stimuli with train duration of 1 s), and 60 consecutive auditory pulses with a repetition rate of 60 Hz (S3: 60-Hz rapid periodic stimuli with train duration of 1 s). The stimuli were presented at the intensities of 6, 20, and 26 dB above individual sensation level (dB SL) (i.e., I1 = 6 dB SL, I2 = 20 dB SL, and I3 = 26 dB SL; Fig. 1).

### Experimental Design

Subjects were seated in a comfortable chair in a silent, shielded room, and were asked to focus their attention on the occurrence of each stimulus. Stimuli of the same type were presented in three blocks in counterbalanced order between participants. In each block 20 stimuli at each of the three intensities (I1–I3) were presented in random order with a randomized inter-stimulus interval between 8 s and 10 s. This resulted in a total of 180 stimulus trials for each subject (20 trials×3 intensities×3 types). Subjects were prompted by a text cue to verbally report the subjective loudness judgment 3 s after each stimulus, using a numerical rating scale ranging from 0 (no sound) to 10 (unbearable sound).

### EEG Recording

The EEG data were recorded using a 64-channel Brain Products system (pass band: 0.01–100 Hz, sampling rate: 500 Hz), connected to a standard EEG cap according to the international 10–20 system. The left mastoid was used as reference channel, and all channels impedances were kept lower than 10 kΩ. To monitor ocular movements and eye blinks, electro-oculographic (EOG) signals were simultaneously recorded from four surface electrodes, one pair placed over the higher and lower eyelid, the other pair placed 1 cm lateral to the outer canthus of the left and right eye.

### EEG Data Analysis

#### Preprocessing

EEG data were imported and preprocessed using EEGLAB [26], an open source toolbox running under the MATLAB environment. Continuous EEG data were bandpass filtered between 1 and 100 Hz. EEG epochs were extracted using a time window of 3000 ms (1000 ms pre-stimulus and 2000 ms post-stimulus), and baseline corrected using the pre-stimulus time interval. Epoched trials contaminated by eye-blinks and movements were corrected using an Independent Component Analysis algorithm [26]–[28]. In all datasets, independent components with a large EOG channel contribution and a frontal scalp distribution were removed. After Independent Component Analysis and additional baseline correction, EEG trials were re-referenced to the bilateral mastoid electrodes.

#### Time domain analysis

For each subject and each condition (3 stimulus types×3 stimulus intensities), artifact-free EEG epochs were averaged to attenuate the contribution of activities non-phase-locked to the onset of the stimulus. The obtained averaged waveforms were lowpass filtered at 30 Hz (zero-phase digital filtering) [29] to emphasize the ERPs evoked by the onset of auditory stimuli. For S1, the averaged waveforms were bandpass filtered between 35 and 45 Hz and between 55 and 65 Hz (zero-phase digital filtering) to highlight the MLRs around 40 Hz and 60 Hz respectively. For S2 and S3, the obtained averaged waveforms were respectively bandpass filtered between 35 and 45 Hz and between 55 and 65 Hz (zero-phase digital filtering) [29] to extract SSRs, synchronized to 40-Hz and 60-Hz rapid periodic stimulation.

The peak latency and baseline-to-peak amplitude of N1 and P2 were measured at Fz for each subject, and then were compared using a 3-level (stimulus type: S1, S2, S3) one-way repeated-measures analysis of variance (ANOVA) with a statistical significance level of 0.05. When the main effect of the ANOVA was significant, Tukey post hoc multiple comparisons were performed.

The amplitudes of MLRs and LLRs to transient stimulation, as well as SSRs to 40-Hz and 60-Hz periodic stimulation, were measured at Fz for each subject (MLRs [absolute value]: 10–100 ms; LLRs: 110–170 ms; SSRs [absolute value]: 0–1000 ms). The amplitudes at different stimulus intensities were compared using the 3-level (I1, I2, and I3) one-way repeated-measures ANOVA.

#### Time-frequency domain analysis

Time-frequency distributions (TFDs) of auditory-evoked, auditory-induced, and phase-locking value of brain responses elicited by transient and periodic auditory stimulation were calculated using a windowed Fourier transform (WFT) with a fixed 200-ms width Hanning window. Such a time-frequency analysis was selected to achieve a good tradeoff between time resolution and frequency resolution in a wide range of EEG frequencies [30]. It thus yielded a complex time-frequency spectral estimate 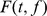 at each point 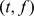 of the time-frequency plane extending from −1000 ms to 2000 ms (in steps of 2 ms) in latency, and from 1 to 100 Hz (in steps of 1 Hz) in the frequency.

For each estimated frequency, TFDs were baseline corrected using the pre-stimulus interval (pre-stimulus −800 to −200 ms) to calculate the relative change of power (expressed as ER%), according to the formula:
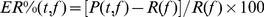
(1)where 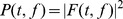 is the power spectral density at a given time-frequency point 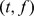, and 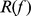 is the averaged power spectral density of the signal enclosed within the pre-stimulus reference interval (−800 to −200 ms before the onset of the stimulation), for each estimated frequency 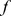.

It should be noted that the auditory-evoked TFDs, which only contained phase-locked neural activities (ERPs), were obtained by performing WFT on the averaged waveform, while the auditory-induced TFDs, which contained both phase-locked (ERPs) and non-phase-locked (event-related synchronization and desynchronization) neural responses, were obtained by performing WFT on single-trial EEG responses and averaging across trial. Furthermore, the phase-locking value (PLV) [31], a measure of phase synchrony across trials, was calculated for each subject and stimulus type, as follows:
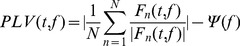
(2)Where N is the number of trials and 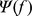 is the average PLV of the pre-stimulus interval (−800 to −200 ms before the onset of the stimulation) for each estimated frequency 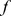.

To test whether the brain responses within the post-stimulus time-frequency region were significantly different from those within the pre-stimulus time-frequency region, a bootstrapping method [26], [32] was performed on the obtained TFDs. For each time-frequency pixel in the post-stimulus interval, investigated populations and reference populations were collected from 19 subjects. The null hypothesis was that there was no mean difference between these two populations. Then pseudo-t statistic between the two populations was calculated, and we estimated the probability distribution of the pseudo-t statistic from the reference population by drawing with replacement two populations of the same size. The permutation was executed for 5000 times. The distribution of the pseudo-t statistics from the reference population and the bootstrap P value for the null hypothesis were generated. This procedure yielded time-frequency regions, in which the TFDs were significantly different relative to the reference region [33], [34]. To address the problem of multiple comparisons, the significance level (P value) was corrected using a false discovery rate procedure [32].

Based on the time-frequency features of LLRs, 40-Hz SSRs, and 60-Hz SSRs, the time-frequency region-of-interest (TF-ROI) was respectively defined as follows: S1: 1–500 ms in latency and 1–10 Hz in frequency; S2: 1–1000 ms in latency and 35–45 Hz in frequency; S3: 1–1000 ms in latency and 55–65 Hz in frequency. Within each TF-ROI, the magnitude of brain responses was estimated by computing the mean of neural activities from all included time-frequency pixels.

To assess the relationship between the scalp topography of transient responses (especially for MLRs) and SSRs, we (1) estimated the scalp topographies of transient responses (S1) from the magnitudes of brain responses for all channels at the time interval of 10–100 ms of each frequency (ranging from 1 to 100 Hz), (2) estimated the scalp topographies of SSRs from the magnitudes of brain responses for all channels within their respective TF-ROI (S2: 1–1000 ms in latency and 35–45 Hz in frequency; S3: 1–1000 ms in latency and 55–65 Hz in frequency), and (3) calculated the correlation coefficients and their significance between the scalp topographies of transient responses at each frequency and those of 40-Hz and 60-Hz SSRs respectively.

To test the relationship between the amplitudes of SSRs and transient responses, we first performed the correlation analysis (1) between N1 power in S1 (the square of N1 amplitude) and 40-Hz steady-state power in S2, (2) between N1 power in S1 and 60-Hz steady-state power in S3, (3) between 40-Hz steady-state power in S2 and 60-Hz steady-state power in S3.

To explore the relationship between auditory-evoked responses and the stimulus intensity as well as the subjective loudness judgment, for each subject and each stimulus type (S1–S3), we first performed a linear regression analysis between the magnitude of time-frequency representation (measured at Fz) and the subjective loudness judgment across all single trials for each time-frequency pixel. This procedure yielded time-frequency distribution of regression coefficient, which coded the strength and direction of the relationship between the magnitude of time-frequency representation and the subjective loudness judgment as a function of time and frequency for each subject. The single-subject time-frequency distribution of regression coefficient was averaged to obtain the group-level time-frequency distribution of regression coefficient. For each subject, the mean of regression coefficients from all included time-frequency pixels in each pre-defined TF-ROI (LLRs: 1–500 ms in latency and 1–10 Hz in frequency; 40-Hz SSRs: 1–1000 ms in latency and 35–45 Hz in frequency; 60-Hz SSRs: 1–1000 ms in latency and 55–65 Hz in frequency) were calculated, and were compared against zero using a one-sample t-test. In addition, to rule out the influence of the variability of stimulus intensity, we removed (1) the mean magnitude of time-frequency representation from single-trial time-frequency representations and (2) the mean rating of subjective loudness judgment from single-trial ratings for each subject and each stimulus intensity, and performed the same linear regression analysis. The mean of regression coefficients from all included time-frequency pixels in the same TF-ROIs were calculated, and were compared against zero using a one-sample t-test.

## Results

### Electrophysiological Results: Time Domain

**Figure 2:**
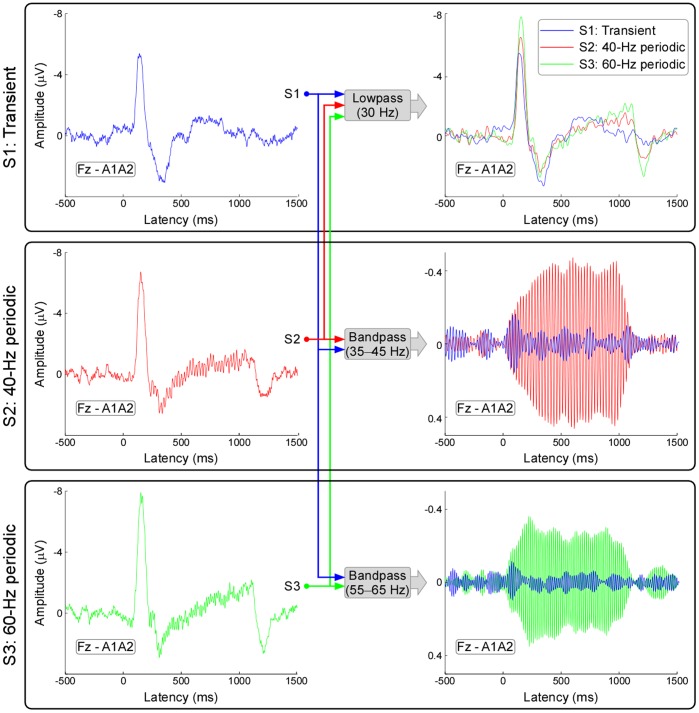
Event-related potentials (ERPs) elicited by transient and periodic auditory stimulation. Waveforms (recorded at electrode Fz) obtained following transient, 40-Hz periodic, and 60-Hz periodic stimulation are shown in blue, red, and green respectively. The onset of both transient and periodic stimulation elicited clear ERPs consisting a dominant negative peak followed by a positive peak after the use of 1–30 Hz bandpass filter. Even with a low SNR, the middle latency responses (MLRs, i.e., gamma band oscillations) were clearly presented around both 40 Hz and 60 Hz at the early latencies (10–100 ms). Steady-state brain responses, synchronized to 40-Hz and 60-Hz periodic auditory stimulation, were clearly presented using a 35–45 Hz and 55–65 Hz bandpass filter respectively.

Fig. 2 displayed the grand averaged ERP waveforms measured at Fz for different stimulus types (S1–S3; from top to bottom). Extracted using a 1–30 Hz bandpass filter, ERPs elicited by the onset of auditory stimuli, showed a dominant negative peak at 145±17 ms, 156±13 ms and 157±14 ms, followed by a clear positive peak at 296±52 ms, 297±55 ms and 294±55 ms (for S1, S2, and S3 respectively). Across subjects, latencies and amplitudes of N1 and P2 were summarized in Table 1, and all these parameters, except N1 latency, were not significantly different across different stimulus types (S1–S3; P>0.05 for all comparisons). Even with a low SNR, the MLRs (i.e., gamma band oscillations) were clearly presented around both 40 Hz and 60 Hz at the early latencies (10–100 ms) (middle right and bottom right panels of Fig. 2). Steady-state brain responses, synchronized to 40-Hz and 60-Hz periodic auditory stimulation, were extracted using a narrow 35–45 Hz and 55–65 Hz bandpass filter for S2 and S3 respectively (middle right and bottom right panels of Fig. 2).

**Table 1:** The steady-state power, and the latency and amplitude of N1 and P2 (± SD) at different stimulus types (S1–S3). Right columns report ANOVA results.

	Stimulus type			ANOVA	
	S1	S2	S3	F	P
Steady-state power (µV^2^)	–	0.31±0.15	0.22±0.13	–	–
N1 latency (ms)	145±17	156±13	157±14	3.520	0.037
N1 amplitude (µV)	−6.47±2.49	−7.12±2.76	−8.40±2.97	2.431	0.098
P2 latency (ms)	296±52	297±55	294±55	0.015	0.985
P2 amplitude (µV)	5.06±2.44	4.85±2.36	4.97±2.47	0.035	0.965

**Figure 3:**
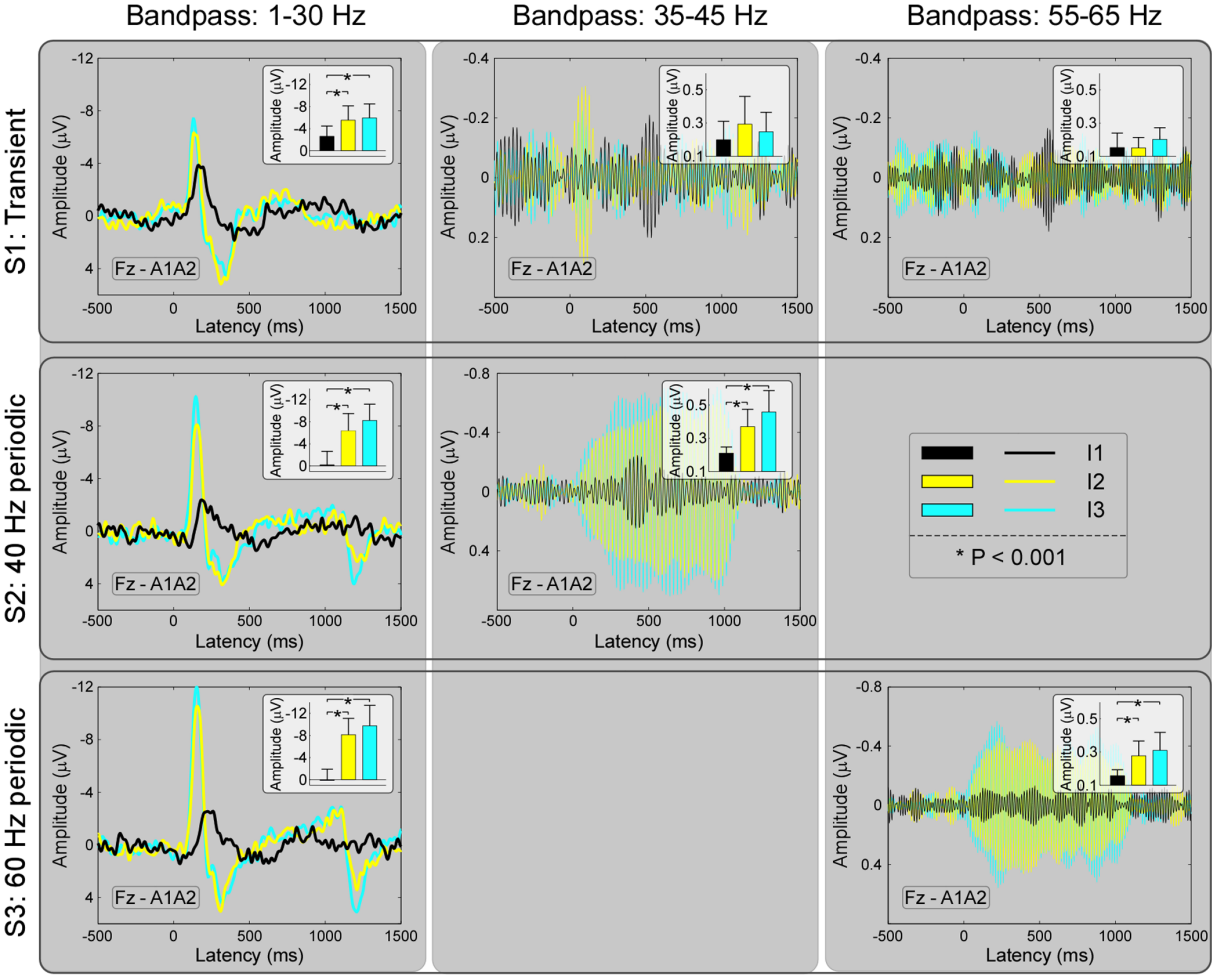
Event-related potentials (ERPs) elicited by transient and periodic auditory stimulation at different stimulus intensities (I1–I3). Waveforms (recorded at electrode Fz) obtained following both transient and periodic auditory stimulation at stimulus intensities of I1, I2, and I3 are shown in black, yellow, and cyan respectively. Amplitudes of late latency responses (LLRs, for S1, S2, and S3) and SSRs (40-Hz for S2 and 60-Hz for S3) were significantly different across different stimulus intensities (I1–I3; P<0.05 for all comparisons), while amplitudes of middle latency responses (MLRs, between 35 and 45 Hz and between 55 and 65 Hz for S1) were not significantly different across different stimulus intensities (I1–I3; P>0.05 for both comparisons).

Across subjects, amplitude of transient responses (MLRs and LLRs) and SSRs (40 Hz and 60 Hz) at different stimulus intensities (I1–I3) in each stimulus type (S1–S3) were summarized in Table 2. As displayed in Fig. 3, amplitudes of LLRs (for S1, S2, and S3) and SSRs (40-Hz for S2 and 60-Hz for S3) were significantly different across different stimulus intensities (I1–I3; P<0.05 for all comparisons), while amplitudes of MLRs (between 35 and 45 Hz and between 55 and 65 Hz for S1) were not significantly different across different stimulus intensities (I1–I3; P>0.05 for both comparisons) (Fig. 3).

**Table 2:** Amplitudes of transient responses and steady-state responses at different stimulus intensities and ANOVA results.

			Stimulus intensity			ANOVA	
			I1	I2	I3	F	P
**Stimulus type**	S1	LLRs (µV)	−2.61±1.92	−5.53±2.67	−5.93±2.62	10.6	<0.001
		MLRs (∼40 Hz, µV^2^)	0.20±0.12	0.29±0.17	0.25±0.12	2.17	0.124
		MLRs (∼60 Hz, µV^2^)	0.15±0.09	0.15±0.07	0.20±0.07	2.77	0.072
	S2	LLRs (µV)	−0.20±2.52	−6.36±3.23	−8.22±3.06	38.46	<0.001
		SSRs (∼40 Hz, µV^2^)	0.21±0.04	0.37±0.11	0.46±0.13	28.98	<0.001
	S3	LLRs (µV)	0.13±2.09	−8.14±3.03	−9.74±3.84	56.43	<0.001
		SSRs (∼60 Hz, µV^2^)	0.16±0.04	0.28±0.09	0.31±0.11	16.83	<0.001

### Electrophysiological Results: Time Frequency Domain

**Figure 4:**
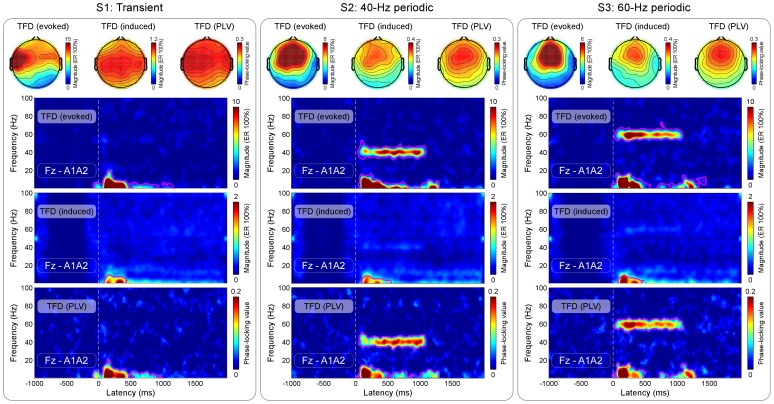
Time-frequency distributions (evoked, induced, and PLV) of brain responses elicited by transient and periodic auditory stimulation. For all types of stimulation, time-frequency distributions (recorded at electrode Fz) of auditory-evoked responses, auditory-induced modulation of EEG oscillatory magnitudes, and PLV are displayed from top to bottom in each panel. x-axis, time (ms); y-axis, frequency (Hz). The color scale represents the average change of the corresponding value to the presentation of stimulation, relative to the pre-stimulus interval. The regions circled by purple lines had significantly larger values than those in the pre-stimulus interval (P≤0.001). Whereas the onset of all stimulation elicited the enhancement of evoked TFDs, induced TFDs, and PLV (1–500 ms in latency and 1–10 Hz in frequency), both 40-Hz and 60-Hz periodic stimulation did so only for evoked TFDs and PLV (around 40 Hz and 60 Hz respectively) (P≤0.001). For each type of stimulation and each type of TFDs, the scalp topography was measured at the corresponding TF-ROI (transient: 1–500 ms in latency and 1–10 Hz in frequency; 40-Hz periodic: 1–1000 ms in latency and 35–45 Hz in frequency; 60-Hz periodic: 1–1000 ms in latency and 55–65 Hz in frequency). Note that scalp topographies elicited by both 40-Hz and 60-Hz periodic stimulation were similarly maximal at frontal region (near Fz), while scalp topographies elicited by transient stimulation were maximal at vertex and bilateral temporal regions.

Fig. 4 displayed the TFDs (measured at Fz) of auditory-evoked, auditory-induced, and PLV of brain responses elicited by transient and periodic auditory stimulation (from top to bottom of each panel). The time-frequency regions with significantly larger values than those in the pre-stimulus reference interval, were circled with white lines (P≤0.001). Whereas the onset of all stimulation elicited the enhancement of evoked TFDs, induced TFDs, and PLV (1–500 ms in latency and 1–10 Hz in frequency), both 40-Hz and 60-Hz periodic stimulation did so only for evoked TFDs and PLV (around 40 Hz and 60 Hz respectively) (P≤0.001). For each type of stimulation and each type of TFDs, the scalp topography, measured at the corresponding TF-ROI, was presented on the top of Fig. 4. Note that the scalp topographies elicited by both 40-Hz and 60-Hz periodic stimulation were similarly maximal at frontal region (near Fz), while the scalp topographies elicited by transient stimulation were maximal at vertex and bilateral temporal regions.

**Figure 5:**
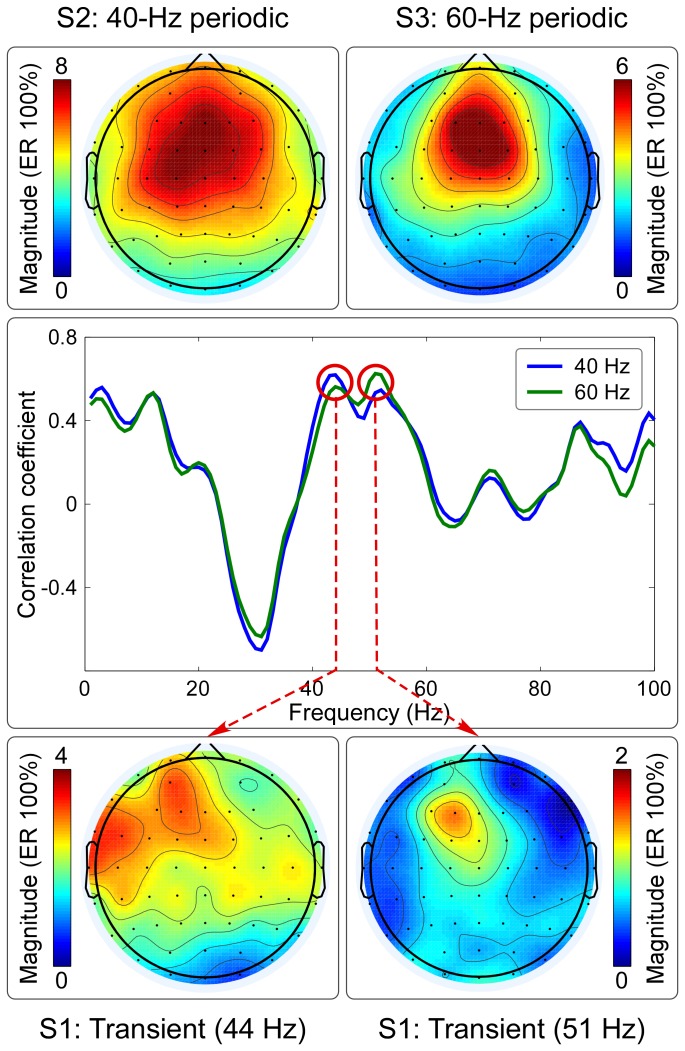
The relationship between the scalp topographies of transient responses and steady-state responses. The correlation coefficients between the scalp topographies of transient responses at the time interval of 10–100 ms of each frequency (ranging from 1 to 100 Hz) and those of 40-Hz and 60-Hz SSRs are respectively showed in blue and green lines (middle panel). The frequencies showing the highest correlation coefficients between transient responses and 40-Hz and 60-Hz SSRs were respectively observed at 44 Hz and 51 Hz, which are marked using red circles (middle panel). The scalp topographies of 40-Hz and 60-Hz SSRs showed a similar maximum at frontal region (near Fz) (top left and top right panels respectively). Even with a low SNR, the scalp topographies of transient responses at 44 Hz and 51 Hz also showed a similar maximum at frontal region (near F1) (bottom left and bottom right panels respectively).In Fig. 6, we showed that no significant correlation was observed when examining the relationship between N1 power and 40-Hz steady-state power in S2 (top left panel of Fig. 6; R = −0.08, P = 0.74), as well as between N1 power and 60-Hz steady-state power in S3 (top middle panel of Fig. 6; R = −0.01, P = 0.97). In contrast, significant correlation was observed when examining the relationship between 40-Hz steady-state power in S2 and 60-Hz steady-state power in S3 (top right panel of Fig. 6; R = 0.71, P<0.001).

Fig. 5 showed that the highest correlation coefficients between scalp topographies of transient responses (especially for MLRs) and of 40-Hz and 60-Hz SSRs were similarly maximal at 44 Hz and 51 Hz (R = 0.62, P<0.05 and R = 0.56, P<0.05 for 40-Hz and 60-Hz SSRs respectively at 44 Hz; R = 0.55, P<0.05 and R = 0.63, P<0.05 for 40-Hz and 60-Hz SSRs respectively at 51 Hz) (Fig. 5). Even with a low SNR, scalp topographies at gamma band (44 Hz and 51 Hz, MLRs) elicited by transient stimulation were similarly maximal at frontal region (near F1).

In Fig. 7, we showed the mean of regression coefficients across subjects for LLRs (0.15±0.09; P<0.001, one-sample t-test), 40-Hz SSRs (0.17±0.15; P<0.001), and 60-Hz SSRs (0.13±0.18; P = 0.006) within their respective TF-ROIs (LLRs: 1–500 ms in latency and 1–10 Hz in frequency; 40-Hz SSRs: 1–1000 ms in latency and 35–45 Hz in frequency; 60-Hz SSRs: 1–1000 ms in latency and 55–65 Hz in frequency). After removing the mean magnitude of time-frequency representation from single-trial time-frequency representations for each subject and each stimulus intensity, the mean of regression coefficients across subjects for LLRs, 40-Hz SSRs, and 60-Hz SSRs were respectively 0.05±0.08, −0.03±0.13, and −0.02±0.14. Note that the regression coefficients for LLRs were still significantly and positively correlated with the subjective loudness judgment (P = 0.02), but not for 40-Hz and 60-Hz SSRs (P = 0.31 and P = 0.53 respectively). These results indicated that SSRs were dominantly modulated by stimulus intensity, but not modulated by subjective loudness judgment within the same stimulus intensity. In contrast, LLRs were not only strongly modulated by stimulus intensity, but also significantly modulated by subjective loudness judgment within the same stimulus intensity.

## Discussion

**Figure 6:**
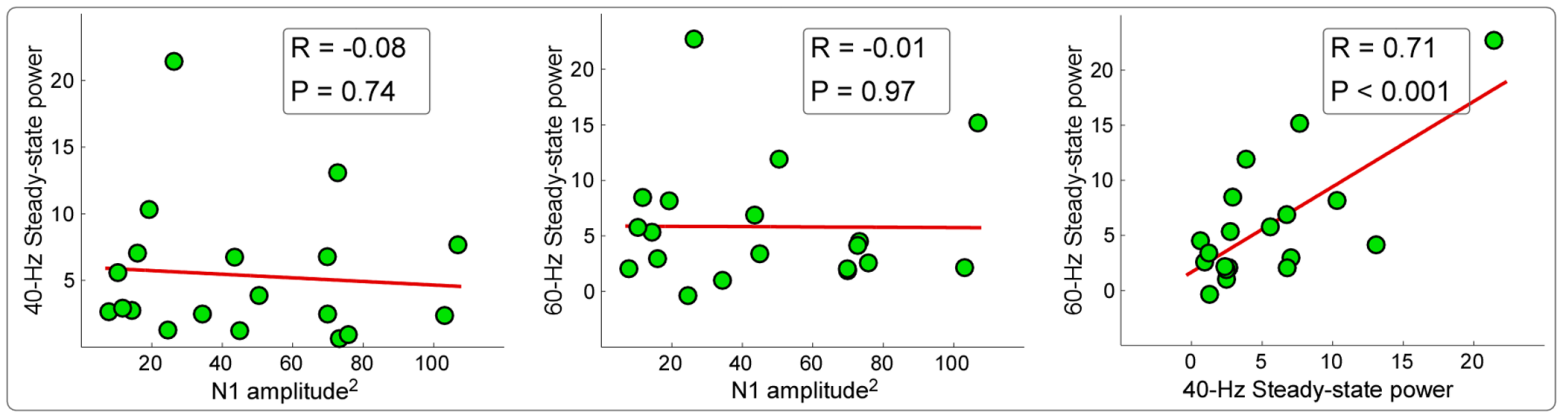
Correlations of power between transient responses and steady-state responses. No significant correlation was observed when examining the relationship between N1 power and 40-Hz steady-state power in S2 (left panel; R = −0.08, P = 0.74), as well as between N1 power and 60-Hz steady-state power in S3 (middle panel; R = −0.01, P = 0.97). In contrast, significant correlation was observed when examining the relationship between 40-Hz steady-state power in S2 and 60-Hz steady-state power in S3 (right panel; R = 0.71, P<0.001). Each green point represents one subject, and the red lines represent the best linear fit.

**Figure 7:**
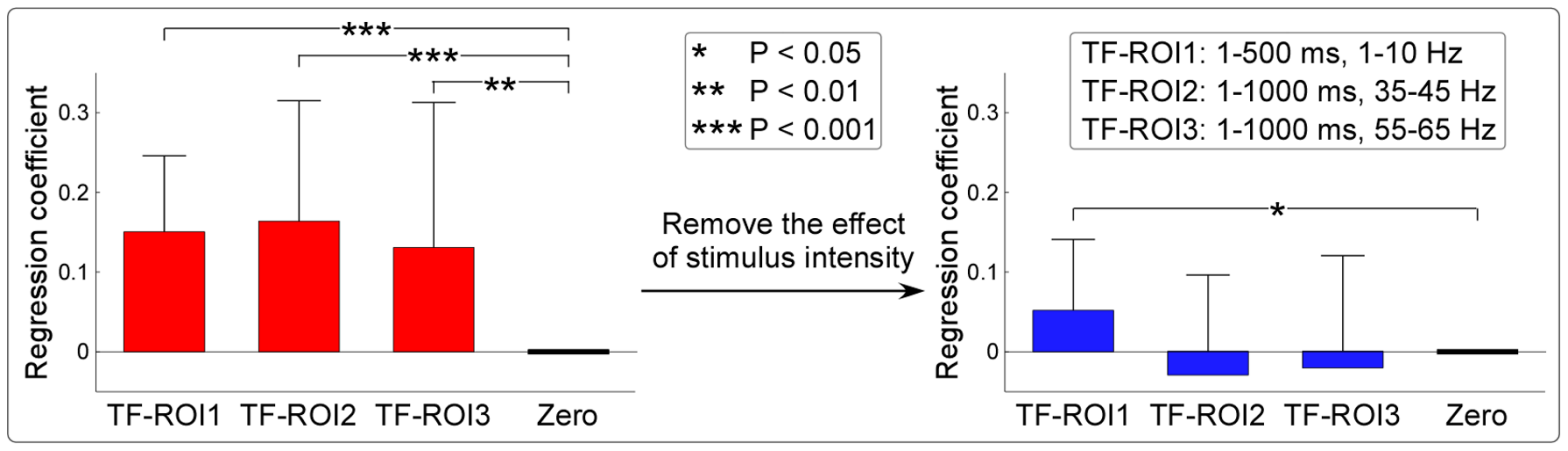
Relationship between the subjective loudness judgment and brain responses elicited by transient and periodic auditory stimulation. The mean of regression coefficients across subjects for LLRs, 40-Hz SSRs, and 60-Hz SSRs within their respective TF-ROIs were all significantly larger than zero (P<0.001, P<0.001, and P = 0.006 respectively; left panel). After removing the mean magnitude of time-frequency representation from single-trial time-frequency representations for each subject and each stimulus intensity, the mean of regression coefficients across subjects for LLRs was still significantly larger than zero (P = 0.02), but for 40-Hz SSRs, and 60-Hz SSRs were not (P = 0.31 and P = 0.53 respectively) (right panel). Error bar represents the standard deviation (SD) of regression coefficients across subjects for each condition.

In this study, taking auditory modality as an example, we systematically examined the relationship between transient ERPs and SSRs. We obtained the following five findings: (1) the amplitudes of LLRs in transient ERPs and SSRs were similarly and significantly different at different stimulus intensities, while amplitudes of MLRs in transient ERPs, even showing similar temporal and spectral features with SSRs, were not significantly different (Figs. 2–3); (2) whereas significant power enhancement of LLRs in transient ERPs was observed at both single-trial and averaged levels, which was not observed for MLRs in transient ERPs, significant enhancement of SSR power was only observed at the averaged level, thus confirming the important contribution of phase synchronization, but not power enhancement, to the generation of SSRs (Fig. 4); (3) Scalp topographies of LLRs in transient ERPs (maximal at vertex and bilateral temporal regions) were markedly different from those of SSRs (maximal at frontal region), while scalp topographies of MLRs in transient ERPs were similar with those of SSRs (maximal at frontal region), even with a low SNR (Figs. 4–5); (4) the powers of both 40-Hz and 60-Hz SSRs were significantly correlated, while they were not significantly correlated with N1 power in transient ERPs (Fig. 6); (5) the amplitude of SSRs was significantly modulated by stimulus intensity, but not significantly modulated by subjective loudness judgment within the same stimulus intensity, while the amplitude of LLRs in transient ERPs was not only significantly modulated by stimulus intensity, but also significantly modulated by subjective loudness judgment within the same stimulus intensity (Fig. 7). All these findings indicated that SSRs at high frequencies (e.g., 40 and 60 Hz) were markedly different with MLRs and LLRs in transient ERPs. SSRs at high frequencies (e.g., 40 and 60 Hz) were not likely generated from linear superposition of series of MLRs and LLRs in transient ERPs using the presented experimental paradigm (Fig. 1). Therefore, along with the high SNR nature, SSRs, represented as distinct neural responses from transient ERPs, could be of great importance to study neural mechanisms of the human brain in both basic and clinical studies.

Since Regan firstly described the recording of SSRs as an alternative approach to investigate the neural activities in EEG [35], an increasing number of studies have used SSRs to explore the neural activities evoked by periodic repetition of various sensory stimuli (e.g., visual, auditory, somatosensory, and nociceptive) [13]–[15], [36]. However, we are not clear about how SSRs emerge from EEG, and it still remains a debate about the relationship between SSRs and transient ERPs [5]. As one of the most popular theories, superposition hypothesis suggested that SSRs evoked by periodic repetition of sensory stimuli were composed of the linear summation of successive transient ERPs evoked by single sensory stimulus [14]–[18], [24], [37]. This hypothesis was normally supported by the evidence that SSRs, evoked by high-frequency auditory and visual stimuli, were consistent with the waveforms synthesized by linear summation of transient ERPs [14]–[18], [24], [37]. According to this hypothesis, SSRs and transient ERPs reflected the same neural activities, and represented the same underlying neural mechanisms [14]–[18], [24], [37]. This superposition hypothesis was challenged by the oscillatory entrainment hypothesis, which indicated that SSRs reflected the entrainment or resonance of a population of neurons responding to the periodic repetition of sensory stimuli [19]–[23]. According to this hypothesis, SSRs and transient ERPs should reflect distinct neural activities that may be generated from different neural sources, thus representing different underlying neural mechanisms [19]–[23].

To clarify the relationship between SSRs and transient ERPs, the temporal, spectral, and spatial characteristics of SSRs evoked by 40-Hz and 60-Hz periodic auditory stimulation were studied and compared with those of transient auditory ERPs elicited by a single click. SSRs at high frequencies (e.g., 40 and 60 Hz) were greatly different with LLRs in transient ERPs, and not likely generated from linear superposition of series of MLRs in transient ERPs for the following reasons.

First, we observed that the scalp topographies of LLRs and SSRs were respectively maximal at vertex and bilateral temporal regions and at frontal region (near Fz) (Fig. 4). However, even with a low SNR, scalp topographies of MLRs were maximal at frontal region, which were similar with those of SSRs (Fig. 5). These findings were confirmed by the observations from source analysis [23], [38], which suggested that SSRs and LLRs to auditory stimulation were generated from different parts of the auditory cortex. SSRs were generated from the primary auditory cortex, which was more anterior and media compared to the sources of N1 in transient ERPs (lateral parts of Heschl's gyrus and the planum temporale) [38].

Then, similar with previous studies [22], [23], we observed significant correlation between powers of 40-Hz and 60-Hz SSRs, but not between powers of SSRs and N1 in transient ERPs (Fig. 6). This result indicated similar power variation of 40-Hz SSRs and 60-Hz SSRs across subjects (i.e., the subject with high 40-Hz steady-state power would have a high 60-Hz steady-state power, and vise versa). In contrast, both 40-Hz and 60-Hz SSRs were not co-varied with the N1 power to transient stimulation (i.e., the subject with high 40-Hz/60-Hz steady-state power cannot imply a high power of transient ERPs, and vise versa).In the present study, time-frequency analysis revealed that the PLV was observed to mimic the evoked TFDs for both transient ERPs and SSRs (Fig. 4), while the induced TFDs did not show any significant power enhancement for SSRs (Fig. 4). These findings indicated that SSRs were largely contributed by the phase synchronization of the ongoing EEG activities without large contributions from the new power evoked by the sensory stimuli [39], [40]. In contrast, the generation of LLRs in transient ERPs was mostly caused by the newly evoked power. The importance of phase synchronization to the generation of SSRs may indicate that periodic repetition of sensory stimuli could introduce the reconstruction of brain oscillatory network, which was represented as the reorganization of the phase of the ongoing EEG activities [39], [40]. Therefore, SSRs, mainly contributed by phase synchronization, may express distinct neural responses from transient ERPs that were mainly contributed by power enhancement.

Last, LLRs and SSRs were significantly different at different stimulus intensities (Fig. 3), and significantly and positively correlated with the subjective loudness judgment (Fig. 7), while MLRs were not significantly modulated by both stimulus intensity (Fig. 3) and subjective loudness judgment (Fig. 7). After removing the effect of stimulus intensity, LLRs were still significantly and positively correlated with the subjective loudness judgment, while both 40-Hz and 60-Hz SSRs were not. These results indicated that (1) SSRs were dominantly modulated by stimulus intensity; (2) LLRs were significantly modulated by subjective loudness judgment even within the same stimulus intensity; (3) MLRs were not significantly modulated by both stimulus intensity and subjective loudness judgment, which may be caused by the low SNR of the MLRs. Therefore, even the variance in brain responses contributed by the stimulus intensity and the variance contributed by the subjective loudness judgment cannot be entirely separated, we provided evidence showing that, compared with SSRs, LLRs were markedly more related to the subjective loudness judgment.

It should be noted that the validity of superposition hypothesis was highly depended on the definition of "transient responses" [15]. If the transient responses were defined as the brain potentials evoked by isolated and infrequent stimulation, and enough time should be provided to the sensory system to return to its resting state before the onset of next stimulus [4], [41]. In this case, a long and variable inter-stimulus interval was normally required to make sure the sensory system has returned to the resting state and to elicit the transient responses, even this requirement has been commonly violated [4]. Based on this definition of transient responses, a poor reconstruction of SSRs from the linear superposition of transient responses [15], [42] was normally observed. However, as mentioned in Capilla et al [15], transient responses can also be defined as the brain potentials evoked by a single event, either isolated or embedded in a stimulus train. In this case, a jittered sequence with a mean stimulation frequency close to the frequency of SSRs could be used to isolate the transient responses, which were frequency specific. Based on this definition of transient responses, a linear relationship between SSRs and frequency specific transient responses was normally observed, thus supporting the superposition hypothesis [15]. The present study did not aim to clarify the discrepancy of the definition of “transient responses”, while it should be mentioned that all results and conclusions of the present study were based on the first definition of “transient responses”.

In conclusion, our results indicated that SSRs at high frequencies (e.g., 40 and 60 Hz) could not be explained by linear superposition of series of transient ERPs using the present experimental design. These findings indicated that SSRs could reflect neural responses distinct from transient ERPs, and captured higher SNR compared to MLRs. Such understanding would be of great importance in both basic and clinical studies since it provided solid base for the application of SSRs, as a new window for us, to reveal novel and reliable neural mechanisms of the human brain.
